# Design and synthesis of quorum-sensing agonist for improving biofilm formation and the application of *Acidithiobacillus thiooxidans* in bioleaching

**DOI:** 10.3389/fmicb.2024.1465633

**Published:** 2024-10-15

**Authors:** Deping Tang, Yanpeng Xi, Wentao Song, Mengjiao Li, Yali Liu, Yanyan Lin, Ran Zhang, Aihong Mao

**Affiliations:** ^1^School of Biological and Pharmaceutical Engineering, Lanzhou Jiaotong University, Lanzhou, China; ^2^Gansu Provincial Academic Institute for Medical Research, Lanzhou, China

**Keywords:** quorum sensing agonist, *Acidithiobacillus thiooxidans*, biofilm, adhesion, bioleaching

## Abstract

**Introduction:**

Currently, there are few investigations on the effect of a synthetic exogenous quorum sensing (QS) agonist on the bioleaching rate of *Acidithiobacillus thiooxidans* (*A. thiooxidans*).

**Methods:**

We created AHL (N-acyl-homoserine lactone) analogues and investigated their effects on *A. thiooxidans* biofilm formation, adsorption kinetics, bioleaching, and mechanism.

**Results:**

The findings revealed that N-(3-thiolactone)- dodecylamine (Y3) significantly increased the biofilm formation of *A. thiooxidans* in 96-well plates and sulfur sheets. Adsorption tests revealed that Y3 increased the adhesion rate, adsorption constant, and adsorption efficiency. Bioleaching tests indicated that Y3 boosted bioleaching efficiency, with Ni^2+^ and Cu^2+^ bioleaching rates increasing by 49.13% and 33.03%, respectively. Transcriptomic analysis revealed that Y3 increased genes associated with QS pathways and biofilm formation, particularly *afeI*, which was dramatically elevated 42 times.

**Discussion:**

The study laid the groundwork for a better understanding of the mechanics of *A. thiooxidans* biofilm formation, which could help improve the potential application of *A. thiooxidans* in bioleaching.

## 1 Introduction

Bioleaching (Hydrometallurgy) is a potential technology for extracting low-grade minerals due to its ease of use, environmental friendliness, and minimal capital requirements (Zhao et al., 2015a; Watling, 2016). It is frequently employed to facilitate the dissolution of low-grade minerals that are characterized by a complex composition (Zhang et al., 2024). *Acidithiobacillus thiooxidans* (*A. thiooxidans*) is widely used to extract metals from low-grade sulfide ores and mine tailings (Yang et al., 2019). The bioleaching rate is close to 45% in column flotation of low-grade sulfide copper ore (Wang et al., 2014). Nevertheless, there remain numerous outstanding issues that require further attention. It is well known that the poor leaching rate, bacterial cultivation and domestication, and long leaching periods are key obstacles in the field of microbial leaching (Ji et al., 2022).

Quorum sensing (QS) plays a pivotal role in the evolutionary process, particularly in the context of interspecies and even interdomain signal transduction (Montgomery et al., 2013). It widely appears in bacteria (extreme or pathogenic microbes) (Cui and Harling, 2005), plants (Castillo-Juárez et al., 2015), and viruses (Kai, 2018; Duddy and Bassler, 2021). For instance, *A. thiooxidans* has been reported to produce 3-O-C8-AHL (Ruiz et al., 2008). The strain could induce N-acyl-homoserine lactone (AHL), which also indicates the existence of alternative pathways involved in AHL biosynthesis and sensing in this bacterium (Bellenberg et al., 2014). It is also confirmed that *A. thiooxidans* has one known QS system. In addition, the external addition of C8-AHL, 3-oxo-C8-AHL, or C10-AHL can strengthen biofilm formation on sulfur tablets (Bellenberg et al., 2014). It has been proposed that natural AHLs may enhance the bioleaching process by increasing adhesion to sulfur and pyrite (González et al., 2013). The artificial AHLs could also facilitate biofilm formation and adsorption of sulfur sheets (Mamani et al., 2016). However, the natural AHLs are susceptible to degradation by quorum-quenching (QQ) enzymes and have a relatively short half-life (McInnis and Blackwell, 2011).

Developing natural ligand analogs or simulating self-inducers is one of the strategies to enhance QS, which can diffuse into adjacent cells and promote the QS function, thereby facilitating biofilm formation. Previous studies reported the kinetic changes of ore adsorption by more bacteria and the adsorption behavior of bacteria on different energy substrates (Erbil, 2011). Some studies have demonstrated that the growth kinetics model of *A. thiooxidans* on sulfur surfaces can quantify the number of cells in the initial exponential stage and the bacterial growth in the subsequent linear stage (Konishi et al., 1995). Feng et al. (2015) undertook a systematic study of the enhanced mechanism of adaptive adsorption behavior during *A. thiooxidans* bioleaching from a mineralogical perspective. Adaptive evolution was found to greatly stimulate and accelerate the adsorption behavior of attached cells, thereby further improving the efficiency of bioleaching. In accordance with the perspective put forth by Li et al. (2018), the formation of biofilms is found to be positively correlated with the efficiency of bioleaching. What sets this study apart is that we attempted to explore the change in cell adsorption behavior of AHL analogs to sulfur flakes and synthesize signal molecule analogs for the biofilm formation in *A. thiooxidans*, with the aim of enhancing bioleaching.

This study aims to investigate the impact of exogenous QS regulators on the biofilm formation, adsorption, and leaching efficiency of *A. thiooxidans*, with a particular focus on the quorum-sensing-mediated leaching of microorganisms.

## 2 Materials and methods

### 2.1 Bacterial strains and culture conditions

#### 2.1.1 Bacterial strains

*A. thiooxidans* BY-02 was grown at 30°C and 150 rpm in Starkey medium [0.2 g·L^−1^ (NH_4_)_2_SO_4_, 3.0 g·L^−1^ K_2_HPO_4_, 0.35 g·L^−1^ CaCl_2_·H_2_O, 0.5 g·L^−1^ MgSO_4_·7H_2_O, 0.01 g·L^−1^ FeSO_4_·7H_2_O, 10 g·L^−1^ sulfur (S^0^) powder, and pH 2.0] (Zhu et al., 2012). Bacteria were collected from the sample through centrifugation at 9000 x g for 8 min.

#### 2.1.2 Pentlandite, sulfur sheet, and sulfur flakes

Pentlandite was obtained from Jinchuan Group Ltd., Jinchang, China. The ores were composed of Mg (18.34%), Fe (13.65%), Ni (0.68%), Cu (0.43%), Mn (0.12%), Co (0.03%), and S (3.11%, w/w), respectively. The particles were meticulously ground to a uniform particle size of 75–125 μm for the preparation of the experiments (Tang et al., 2018).

Sulfur sheets: The sulfur powder was melted at 120°C and poured into the cover slide. The cover slide was quickly pressed and cooled at room temperature (González et al., 2013).

Sulfur flakes: This particle size is equal to the ore particle size.

### 2.2 Chemical synthesis of natural signaling molecule and agonist

The study employed drugs purchased from Shanghai Aladdin Biochemical Technology Co., Ltd. The synthesis method was modified from Ganguly et al. (2011). Synthesis of N-(3-cyclobutyrolactone)-decanamide (Y1): The (S)-(−)-*α*- amino group—*γ*-butyrolactone hydrochloride (1 g, substrate 1), was dissolved in dry CH_2_Cl_2_. Decanoyl chloride and triethylamine (TEA) were stirred under ice bath conditions. After 30 min, the mixture was stirred at room temperature for 20 h after being removed from the ice bath. The reaction process was observed using thin layer chromatography (TLC) on a plate. Once the reaction was complete, any unreacted salts and other residues were washed away and removed. The organic phase was collected each time. Finally, the organic phase solution was merged, and the above steps were repeated three times. The mixture was filtered with filter paper to remove MgSO_4_ particles. Finally, a rotary evaporator was used to spin dry at 50°C to obtain a white powdered product, which is the natural signaling molecule Y1. ^1^H NMR (500 MHz, DMSO-d6) is shown in Supplementary Figure S1: ^1^H NMR (500 MHz, DMSO-*d*_6_) *δ* 8.29 (d, *J* = 8.0 Hz, 1H), 4.52 (dt, *J* = 11.0, 8.6 Hz, 1H), 4.33 (td, *J* = 8.8, 1.9 Hz, 1H), 4.19 (ddd, *J* = 10.6, 8.7, 6.5 Hz, 1H), 2.40–2.32 (m, 1H), 2.18 (t, *J* = 7.3 Hz, 1H), 2.16–2.11 (m, 1H), 2.09 (t, *J* = 7.3 Hz, 1H), 1.48 (tt, *J* = 9.6, 4.7 Hz, 2H), 1.24 (s, 12H), 0.86 (t, *J* = 6.7 Hz, 3H), 54%.

The synthesis of agonist N-(3-thiolactone)-danamide (Y2) involves converting the substrate 1 into DL homocysteine thiolactone hydrochloride (1 g), with other treatments such as the above method, and a reaction time of 12 to 14 h. This is the Y2 analog. ^1^H NMR (500 MHz, DMSO-d6) is shown in Supplementary Figure S2: ^1^H NMR (500 MHz, DMSO-d6) *δ* 8.12 (d, *J* = 8.3 Hz, 1H), 4.59 (dt, *J* = 12.6, 7.6 Hz, 1H), 3.38 (td, *J* = 11.4, 5.3 Hz, 1H), 3.27 (dd, *J* = 10.9, 6.7 Hz, 1H), 2.39 (dt, *J* = 12.2, 6.1 Hz, 1H), 2.18 (t, *J* = 7.3 Hz, 1H), 2.12–2.00 (m, 2H), 1.53–1.44 (m, 2H), 1.24 (s, 12H), 0.86 (t, *J* = 6.6 Hz, 3H), 75%.

The synthesis of agonist N-(3-thiolactone)-dodecylamine (Y3) is based on the Y2 agonist. Dodecanoyl chloride is added dropwise while stirring under ice bath conditions, and other treatments are the same as above. The reaction time is 12 to 14 h. This is the Y3 analog. ^1^H NMR (500 MHz, DMSO-d6) is shown in Supplementary Figure S3: ^1^H NMR (500 MHz, DMSO-*d*_6_) δ 8.12 (d, *J* = 8.4 Hz, 1H), 4.59 (ddd, *J* = 12.6, 8.5, 7.0 Hz, 1H), 3.39 (td, *J* = 11.4, 5.4 Hz, 1H), 3.28 (ddd, *J* = 11.0, 7.0, 1.6 Hz, 1H), 2.40 (dddd, *J* = 12.3, 7.0, 5.4, 1.6 Hz, 1H), 2.19 (t, *J* = 7.4 Hz, 1H), 2.11–2.01 (m, 2H), 1.48 (dt, *J* = 7.4, 4.1 Hz, 2H), 1.25 (s, 16H), 0.89–0.83 (m, 3H), 65%.

**Figure 1:**
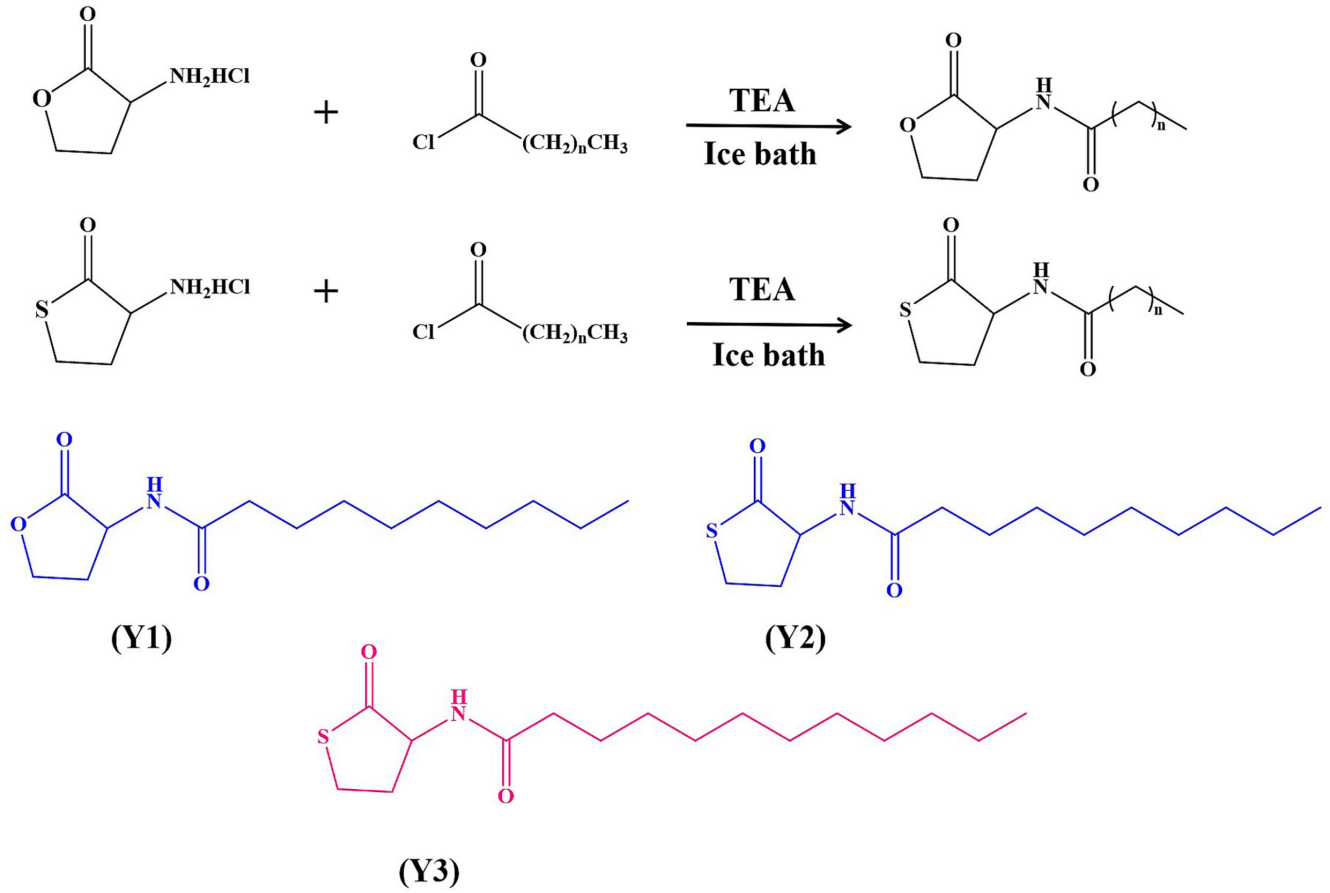
Chemical synthesis of Y1, Y2, and Y3.

Three signal molecule agonists, Y1–Y3, were synthesized by modifying either the inner ester ring or the acyl side chain of the signal molecule, or both simultaneously, with yields all above 54%, as shown in Figure 1. After determining the structures of all pure products using ^1^H NMR, they were dissolved in 0.4% DMSO and stored at −20°C.

### 2.3 Biofilm experiment

Biofilm formation in a 96-well plate was measured employing crystal violet (CV) staining (Zhao et al., 2015b; Tang et al., 2018). A 1.0 × 10^8^ cells/mL *A. thiooxidans* suspension (resuspended in Starkey medium without sulfur powder) containing 0, 10, 50, 100, 150, 200, and 300 μM AHL analogs (Y1, Y2, Y3), respectively, was prepared. 150 μL of cell suspension was added to the 96-well plate (non-pyrogenic, polystyrene). The plate was then incubated at 30°C without agitation for 72 h. After incubation, the biofilm was washed twice with sterile water to remove free bacteria. The plate was stained with 0.5% CV for 15 min, washed twice with sterile water to remove uncombined CV, and then treated with 150 μL of 33% acetic acid. The OD_570_ was measured using an Automatic Microplate Reader (REN90003, America). Each group was performed in 12 replicates. The growth curve was measured by detecting OD_600_.

Biofilm formation on sulfur sheets was observed by scanning electron microscope (SEM) (ZEISS GeminiSEM 500, Carl Zeiss, Germany). A 1.0 × 10^8^ cells/mL *A. thiooxidans* suspension (resuspended in Starkey medium without sulfur powder) containing 300 μM Y1, Y2, and Y3 and without AHL analogs, respectively, was prepared. Then, 1 mL of the bacterial suspension and sulfur sheets were added to a 24-well plate. After being incubated at 30°C without agitation for 3 days, the sulfur sheets were fixed overnight utilizing a glutaraldehyde solution. Subsequently, they were washed twice with PBS buffer and dehydrated with gradient ethanol for 10 min (Brandão et al., 2018). Finally, the biofilm was freeze-dried for 24 h and observed utilizing SEM.

### 2.4 Adsorption experiments

To provide further evidence of the impact of the synthetic agonist Y3 on the adhesion behavior of *A. thiooxidans*, the alteration in the quantity of unoccupied cells indicates the adsorption kinetics of sulfur growth by the bacteria. The number of attached cells was determined by subtracting the planktonic cells from the initial cell count (Zhu et al., 2012). The attachment rate was calculated using the formula: (initial number of free bacterial cells − number of free bacterial cells after adsorption) / initial number of free bacterial cells × 100%. Cell counts were obtained using a blood cell counting board with a depth of 0.1 mm and an area of 1/400 mm^2^. Activated *A. thiooxidans* were passaged three times and cultured until the logarithmic phase was reached. The bacterial solution was collected and inoculated into a fresh shaking bottle with a 10% inoculation amount containing the completed sulfur (2%) flakes as the energy source for *A. thiooxidans* (Harneit et al., 2006). Then, 50, 150, and 300 μM of Y3 were added, with three parallel samples in each group, and the control group was subjected to the same biofilm experiment. Counts were taken, respectively, using a blood cell counter at 0, 20, 40, 60, 80, 100, and 120 min. The adsorption equation is described by Song et al. (2010); Xia et al. (2013); Liu et al. (2019) as follows:


(1) $$\frac{1}{X_{E}}=\frac{1}{X_{M}•K_{A}}•\frac{1}{C_{L}}+\frac{1}{X_{M}}$$

This Equation (1) is a linearized Langmuir adsorption equation, where X_M_ is the maximum adsorption unit mass (cells·g-1), X_E_ represents the concentration of the adsorbed substance onto the ore, and C_L_ denotes the concentration of free cells at equilibrium. K_A_ is the Langmuir adsorption equilibrium constant (g·cells-1), and X_M_ and K_A_ are determined by 1/X_E_ and 1/C_L_ (Liu et al., 2019; Su et al., 2020).


(2) $$\log X_{E}=\log K_{F}+\frac{1}{n}\log C_{L}$$

This Equation (2) is in logarithmic form of the Freundlich equation: K_F_ is the roughly measured area of the attached surface on the solid matrix, reflecting the adsorption capacity; n is an indicator of adsorption efficiency (Xia et al., 2013; Liu et al., 2019).

### 2.5 Bioleaching experiments

The pentlandite (5% w/v in Starkey medium without sulfur powder) was leached by 1 × 10^8^ cells/mL *A. thiooxidans* containing the Y3 of 0, 50, and 150 μM, respectively. The samples were cultured at 30°C and 150 rpm (Xin et al., 2009). The soluble Ni^2+^ and Cu^2+^ concentrations were analyzed periodically by using inductively coupled plasma mass spectrometry (ICP-MS) (Agilent 7,900, American). After 30 days, the ores were washed using distilled water and were dried at 50°C. The surface microstructure of the ores was analyzed using SEM, and the chemical constituents of the surface were determined using energy dispersive spectroscopy (EDS) (Xiong and Guo, 2011; Tang et al., 2018).

### 2.6 Transcriptome experiments

*A. thiooxidans* was cultured in Starkey medium with 0 and 300 μM (Y3), respectively. The bacterial cells were collected and immediately frozen in liquid nitrogen for 10 min before being stored at −80°C. The total RNA was isolated using TRIzol reagent (Invitrogen), and the rRNA was removed using the Ribo-Zero™ rRNA Removal Kit (Epicentre). Clean reads were obtained by removing adapter sequences, low-quality reads (< Q20), and rRNA. These reads were then mapped to *A. thiooxidans* (GCA_001705725.1) using Bowtie2 (http://bowtie-bio.sourceforge.net/bowtie2/index.shtml). Trinity was used for *de novo* transcriptome assembly, RSEM (http://deweylab.github.io/RSEM/) was used for gene expression quantification, and the quantification metric used was TPM (Transcripts Per Million reads). The DESeq2 package, Version 1.24.0, was employed to analyze the differentially expressed genes between samples. To identify significantly differentially expressed genes (DEGs), we used a statistical significance threshold of *p* < 0.05 and a fold change (FC) threshold of >1.5.

This text compares two samples on functional levels using the software Goatools (https://github.com/tanghaibao/GOatools). The analysis employed Goatools Perform GO software for Gene Ontology functional significance enrichment analysis, using Fisher’s exact test. To identify the biological processes most closely related to biological phenomena, KOBAS 2.0 was used to conduct KEGG pathway enrichment analysis. The calculation principle for this analysis is identical to that of GO functional enrichment analysis.

### 2.7 Molecular docking

Molecular docking can be employed to ascertain the binding activity between molecules. This study considers the role of the AfeI/R protein in *A. thiooxidans*, which plays an important role in the quorum-sensing (QS) system (Gao et al., 2020). In combination with the transcriptome results, *afeI* was significantly upregulated. Therefore, the AfeI protein was identified as the target protein for further investigation. The PDB database was consulted to retrieve the ID of AfeI (Entry number: A0A1C2JB12). The ligand was designated as natural Y1 or artificial Y3, and the AutoDock Vina (http://mgltools.scripps.edu, version 1.1.2) molecular docking program was employed for the docking process. The PyMOL software is employed for the visualization of molecular structures and docking complexes. AutoDockTools is employed for the purposes of hydrogenation, charge checking, the specification of atomic type as AD4, the calculation of Gasteiger, and the construction of docking grid boxes for AfeI on a separate basis. Furthermore, the root of the ligand small molecules should be determined in AutoDockTools. The reversible bond of the selected ligand should also be identified. Ultimately, the receptor and ligand molecules must be converted from the “mol2” format to the “PDBQT” format in AutoDockTools for subsequent docking. Following the docking process with Vina, the score for the combination of small molecules and AfeI protein should be calculated. Subsequently, the force analysis and visualization of the docking complex can be conducted using PyMOL and Discovery Studio software.

### 2.8 Statistical analysis

All the results are presented as mean ± standard deviation, and each experiment was repeated three times. *T*-tests were performed using Origin 2021 and SPSS 20.0 to analyze the statistical differences between the control group and the experimental group. A *p-value of* < 0.05 is considered statistically significant.

## 3 Results and discussion

### 3.1 The impact of AHL analogs on biofilm formation and adsorption equilibrium

**Figure 2:**
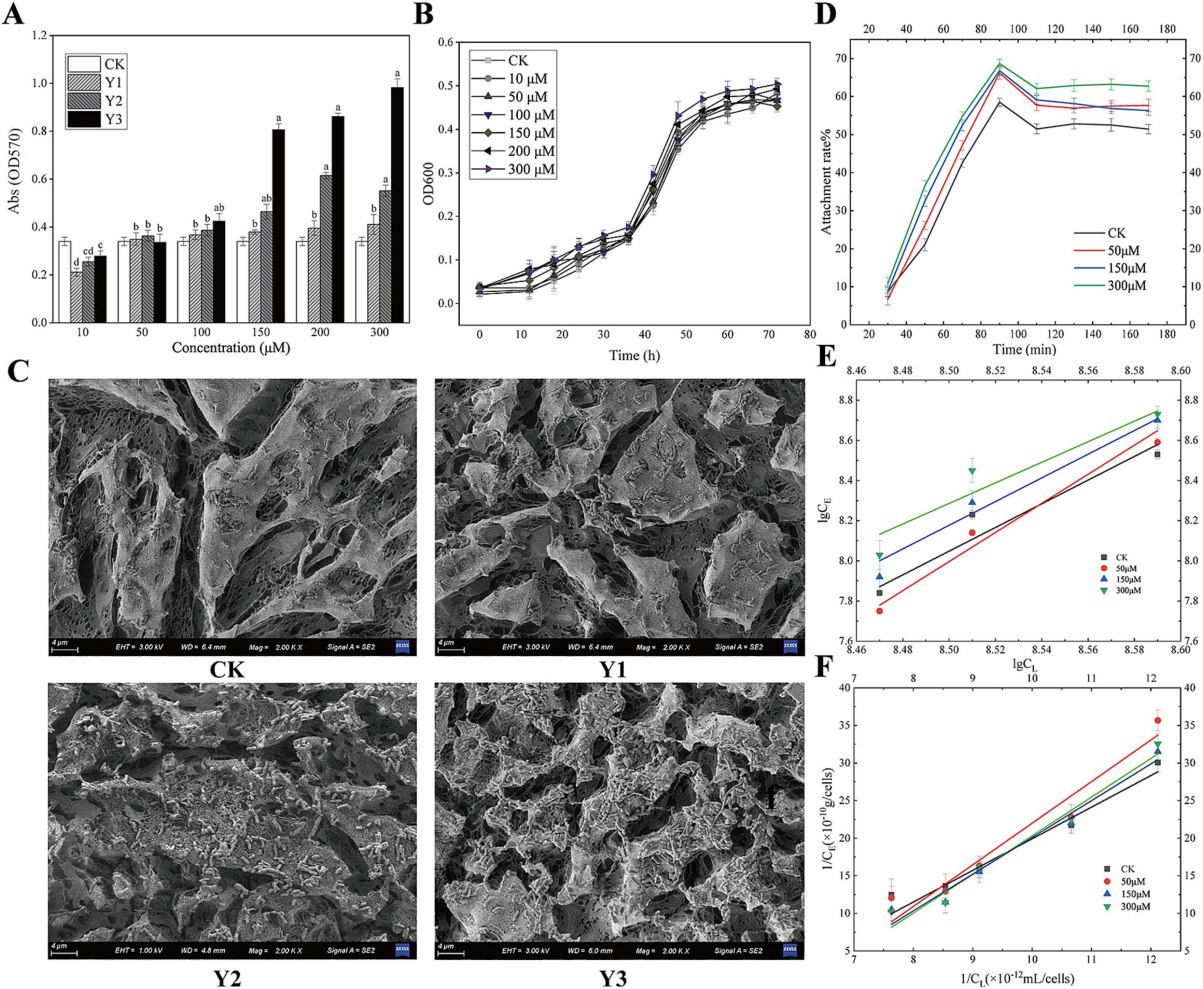
Impact of AHL analogs on *Acidithiobacillus thiooxidans* biofilm formation and adsorption equilibrium. **(A)** The effects of Y1, Y2, and Y3 on the biofilm formation of *Acidithiobacillus thiooxidans* in 96-well plate. **(B)** The effect of Y3 on the growth curve of *Acidithiobacillus thiooxidans*. **(C)** The effects of Y1, Y2, and Y3 on the biofilm formation of *Acidithiobacillus thiooxidans* on sulfur sheets. **(D)** The effect of Y3 on *Acidithiobacillus thiooxidans* attachment. **(E)** The effect of Y3 on *Acidithiobacillus thiooxidans* Langmuir adsorption kinetics. **(F)** The effect of Y3 on *Acidithiobacillus thiooxidans* Freundlich adsorption kinetics.

As shown in Figure 2A, when the concentration of the agonists Y1, Y2, and Y3 is low, it does not reach the bacterial QS threshold, so it does not exert the biological effect. When any of the three agonists with a final concentration of 100 μM were added, the biofilm yield increased compared with the control group. When the final concentration of the agonist was 150 μM, compared with the control group, Y2 and Y3 had significant differences. When the concentration of the agonist is above 100 μM, Y3 promotes biofilm significantly more than the natural signaling molecule Y1, with a promotion rate exceeding 15.4%; Y2, on the other hand, has a promotion rate of 5.3%. It can be seen from Figure 2B that the growth curve of agonist Y3 at different concentrations of *A. thiooxidans* is basically the same. Further experiments should be conducted to investigate whether different concentrations of the agonist have any effect on the growth of the strain as the current results indicate no effect.

Figure 2C shows that, in comparison with the control group, agonists Y2 and Y3 facilitated the adsorption of *A. thiooxidans* on the surface of sulfur sheets. Of the two agonists, Y3 exhibited a particularly pronounced promoting effect, resulting in a notable increase in the degree of erosion on the surface of sulfur sheets.

As shown in Figure 2D, different concentrations of Y3 treatment have varying degrees of promoting effect on the adhesion of sulfur sheets. At approximately 80 min, the adhesion rate reaches its maximum, and from 80 to 110 min, the adhesion rate decreases. After 110 min, the adhesion of different concentrations of Y3 to sulfur sheets basically reaches equilibrium. Figures 2E,F show the linear equation of Freundlich adsorption and Langmuir adsorption, indicating that agonist Y3 with different concentrations enhances the adsorption of sulfur flakes by *A. thiooxidans*. Compared with the other three concentrations, the optimal adsorption efficiency was observed at a concentration of 300 μM (Supplementary Table S1). Consequently, the artificial AHLs Y3 exhibit superior efficacy compared to the natural compound Y1.

### 3.2 The impact of analog Y3 on the leaching efficiency of pentlandite

**Figure 3:**
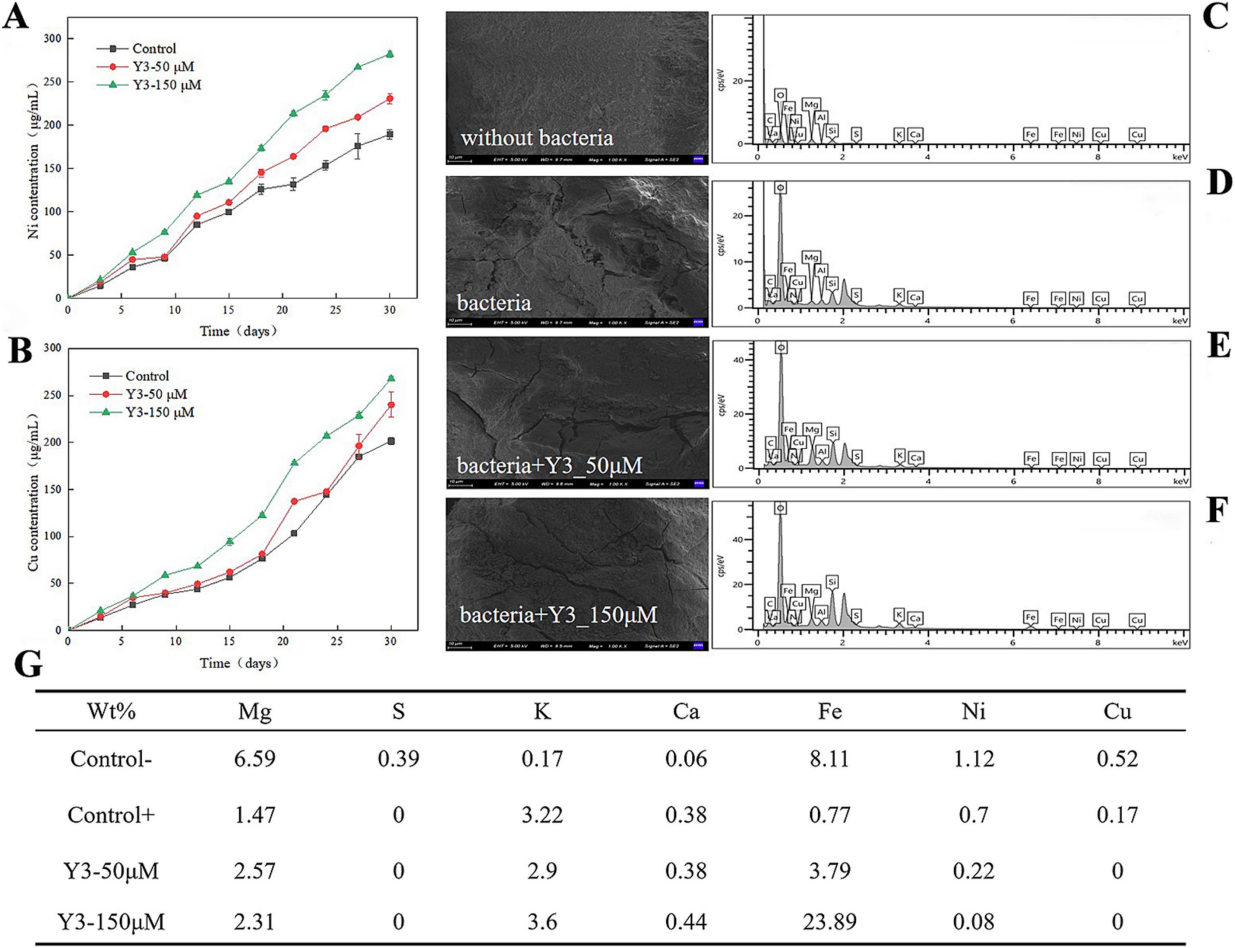
Impact of analog Y3 on the leaching efficiency of pentlandite. **(A)** Ni. **(B)** Cu. **(C–F)** After 30 days of bioleaching, the surface of the ores was analyzed using scanning electron microscopy (SEM) and energy dispersive spectroscopy (EDS). **(G)** The weight percentage of chemical elements on the surface of the ore.

As the leaching time increased, the Y3 treatment resulted in a higher concentration of Ni^2+^ and Cu^2+^ compared to the control group, indicating a positive effect of this agonist Y3 on the leaching of ions by *A. thiooxidans.* The amount of Ni^2+^ and Cu^2+^ leached overall was higher in the Y3 treatment group compared to the control group. After 30 days, the leaching amounts of Ni^2+^ and Cu^2+^ were 282.4 μg/mL and 268.1 μg/mL at 150 μM, respectively. The addition of Y3 promotes the adsorption of bacteria on the pentlandite, enhancing the leaching effect and increasing the Ni^2+^ leaching rate by 49.13%. In addition, the Cu^2+^ leaching rate is increased by 33.03% (Figures 3A,B).

As shown in Figure 3C, the surface of the ore without bacterial addition is smooth and flat, without obvious cracks. After adding the *A. thiooxidans*, the ore surface displayed the appearance of cracks and unevenness, indicating that the bacteria had a certain erosion effect on pentlandite (Figure 3D). Adding 50 μM to the ore treatment revealed more surface cracks (Figure 3E). Increasing the drug concentration further showed that the cracks on the surface of the ore widened and the surface became rougher, indicating that this agonist has a better promoting effect on bacteria in the process of ore treatment (Figure 3F).

*A. thiooxidans* reduces the weight percentage of some chemical elements such as Mg, S, Fe, Ni, and Cu on the surface of the pentlandite, indicating that *A. thiooxidans* helps to free the metals in the leaching process in the form of ions. After adding bacteria and agonist Y3, the S was not detected on the surface of the ore, indicating that the bacteria oxidized S into slightly higher valence polysulfides. The weight percentages of Ni and Cu elements both decreased, indicating that agonist Y3 promoted the leaching of *A. thiooxidans* and released them into the solution, resulting in less residue on the surface of the ore (Figure 3G).

### 3.3 Transcriptome analysis

#### 3.3.1 Extraction and quality of total RNA

As can be seen from Supplementary Table S2, the brightness of RQN ≥ 8.4 RNA 23S is greater than 16S, indicating that RNA is free from impurities such as pigments, proteins, and sugars. The sample concentration (≥ 484 ng/μL) and the total sample requirement (≥ 16 μg) meet the requirements of twice standard construction of prokaryotic transcriptome library, and follow-up experiments can be carried out.

#### 3.3.2 Quality control results of transcriptomic sequencing data

Supplementary Table S3 shows that the average Q20 of the cDNA data is over 95%, indicating that the sequencing quality is high and meets the needs of subsequent analysis. As can be seen from Supplementary Table S4, a total of 35,159,177 reads were obtained by comparing the clean reads of the control group with the designated reference genome. A total of 22,491,429 reads (63.93%) matched with the reference genome, and 21,855,470 reads (62.12%) matched to the unique position of the reference genome. A total of 36,992,859 reads (69.23%) were obtained by comparing the clean reads of the treatment group with the designated reference genome, and 25,669,882 reads matched with the reference genome (69.23%). A total of 24,967,430 reads (67.34%) were matched to the unique location of the reference genome.

**Figure 4:**
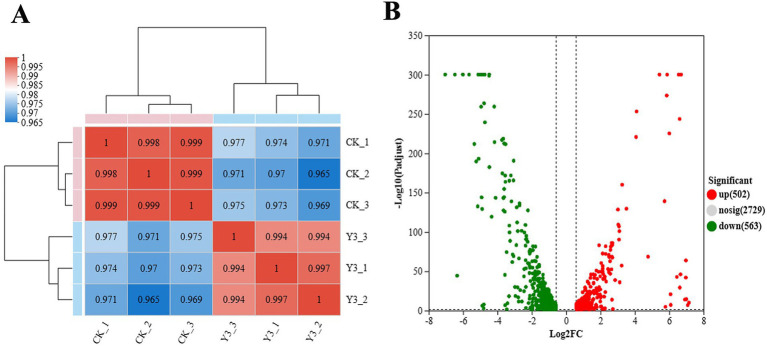
Heat map of expression levels and volcano map of expression differences between samples. **(A)** The right and bottom sides in the figure represent the sample names, while the left and top sides represent the clustering situation of the samples. The different colored squares represent the correlation between the two samples. **(B)** The graph displays the FC value on the horizontal axis, representing the multiple variation value of gene expression differences between two groups of samples. The *p*-value, representing the statistical test value of the difference in gene expression, is shown on the vertical axis.

In this analysis, a total of 3,867 genes were detected, including 3,688 known genes, 106 new genes, and 73 sRNAs. From Figure 4A, the Pearson correlation coefficients within the group are all higher than 0.965, indicating strong sample correlation and good repeatability. The number of upregulation multiples was set to 1.5, and the expression difference volcano diagram in Figure 4B was obtained. There are 502 genes that have been upregulated and 563 genes that have been downregulated.

#### 3.3.3 Functional enrichment analysis

Intergroup differential gene analysis was conducted using DESeq2 software to identify genes with differential expression between the two groups. The screening threshold was set at |log2FC| ≥ 0.585 with P adjust <0.05, based on the quantitative expression results.

**Figure 5:**
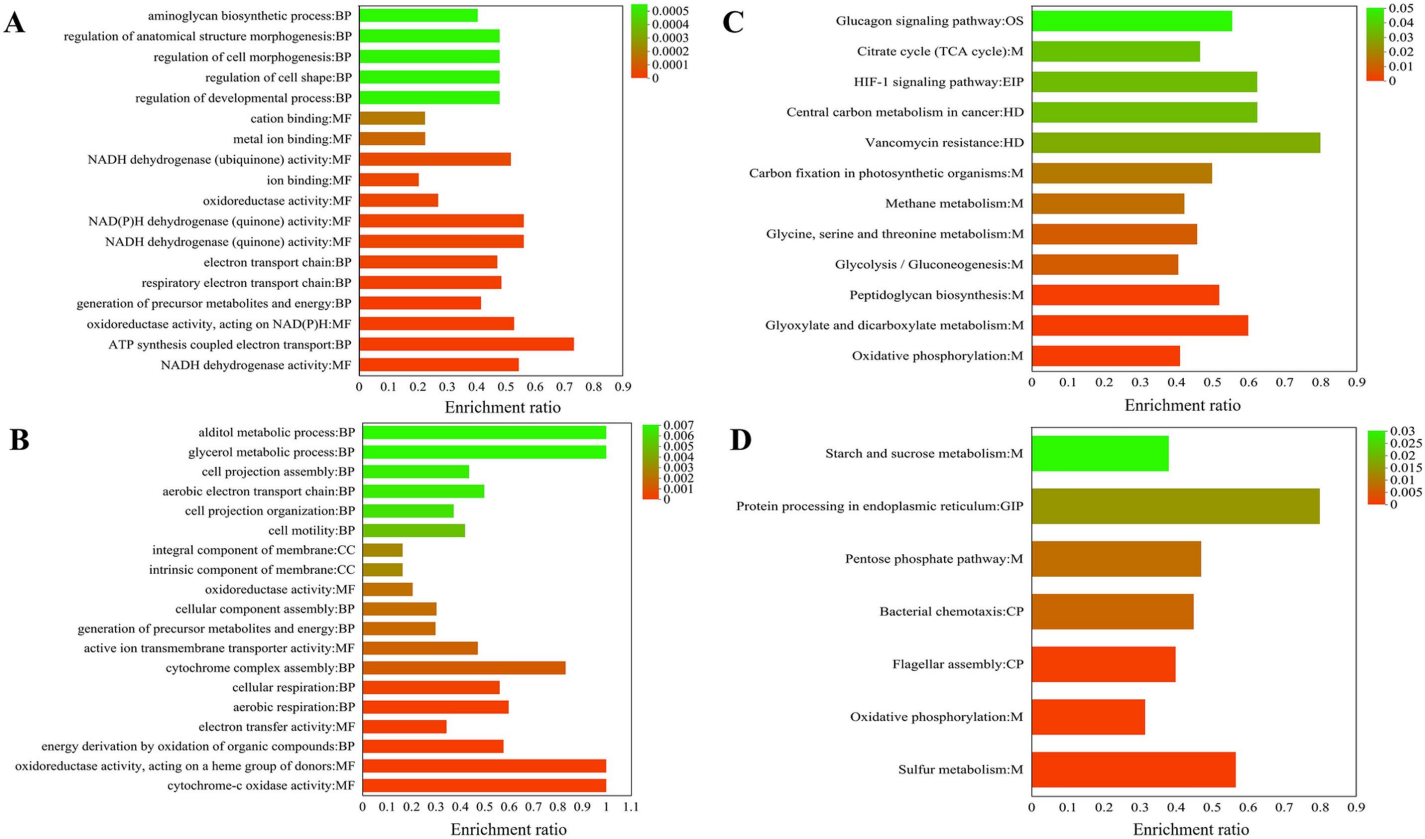
19 most significantly functional enrichment analysis results for all differentially expressed genes (DEGs) are presented. Gene Ontology (GO) enriched analysis upregulated **(A)** and downregulated (**B**); KEGG enriched analysis upregulated **(C)** and downregulated **(D)**.

GO enrichment is divided into three main parts: biological process (BP), cellular component (CC), and molecular function (MF). BP includes ATP synthesis coupled electron transport (11) and generation of precursor metabolites and energy (25), respiratory electron transport chain (17), electron transport chain (17), regulation of developmental process (12), regulation of cell shape (12), regulation of cell morphogenesis (12), regulation of anatomical structure morphogenesis (12), aminoglycan biosynthetic process (15), and glycosaminoglycan biosynthetic process (15). MF mainly includes NADH dehydrogenase activity (18), oxidoreductase activity, acting on NAD(P)H, quinone or similar analogs as acceptor (18), oxidoreductase activity, acting on NAD(P)H (19), NADH dehydrogenase (quinone) activity (18), NAD(P)H dehydrogenase (quinone) activity (18), oxidoreductase activity (63), ion binding (129), NADH dehydrogenase (ubiquinone) activity (14), metal ion binding (73), and cation binding (73) (Figure 5A). High concentrations of hydrogen can also be used for species to produce ATP. This occurs through proton pump activity driven by the ATP of F0-F1-ATP synthase, which serves as an energy source during the reduction of copper ore oxidation (Latorre et al., 2016). As shown in Figure 5B, the NADH pathway belonging to MF and the ATP synthesis pathway of BP are significantly enriched, which facilitates the occurrence of chemical reactions in the acidic environments of *A. thiooxidans.*

Functional enrichment analysis was conducted on all downregulated DEGs. The biological processes (BP) identified were primarily related to energy derivation through the oxidation of organic analogs (11), aerobic respiration (9), cellular respiration (5), cytochrome complex assembly (5), generation of precursor metabolites and energy (18), cellular component assembly (17), cell motility (8), cell projection organization (9), aerobic electron transport chain (6), cell projection assembly (3), glycerol metabolic process (3), and alditol metabolic process (7); CC intrinsic component of membrane (111), integral component of membrane (111), and respirasome (3); MF cytochrome oxidase activity (6), oxidoreductase activity, acting on a heme group of donors (6), electron transfer activity (20), active ion transmembrane transporter activity (9), and oxidoreductase activity (48).

KEGG enrichment is mainly divided into five parts: Environmental Information Processing (EIP), Organismal Systems (OS), Metabolism (M), Cellular Processes (CP), and Human Diseases (HD). Upregulation pathways include the following: The pathways belonging to M include oxidative phosphorylation (30), glyoxylate and dicarboxylate metabolism (15), peptidoglycan biosynthesis (13), glycolysis/gluconeogenesis (13), glycine, serine and threonine metabolism (11), methane metabolism (11), carbon fixation in photosynthetic organisms (8), citrate cycle (TCA cycle) (7); enriched in the HD pathway include vancomycin resistance (4), central carbon metabolism in cancer (5); enriched in the EIP pathway include HIF-1 signaling pathway (5); the OS pathway is glucagon signaling pathway (5) (Figure 5C). Glutamate and cysteine are amino acids that serve as precursors to glutathione, a metabolite that plays a role in the activation of sulfur (Latorre et al., 2016). Surprisingly, in KEGG enrichment map 6c, glyoxylate and dicarboxylate metabolism is significantly enriched, and glutamate synthase (*A6O24_07465*) upregulation is 1.61 (Table 1).

**Table 1:** Statistics of genes related to biofilm formation.

	Gene ID	FC (Y3/CK)	*P*-value	Gene description
Quorum sensing	*A6O24_19840*	42.97	0	N-acyl homoserine lactone synthase
	*A6O24_18640*	2.68	3.40E-32	Radical SAM protein
Biofilm formation (bacterial secretion)	*A6O24_20605*	2.02	2.98E-12	Histidine kinase
	*A6O24_18575*	2.02	2.98E-12	Histidine phosphate transaminase
	*A6O24_04950*	1.68	5.90E-07	Two component sensor histidine kinase
	*A6O24_16560*	1.75	1.41E-06	Two component system response regulator OmpR
Bacterial adhesion	*A6O24_13320*	1.54	4.26E-07	Outer membrane lipid asymmetry maintenance protein MlaD
	*A6O24_17275*	1.92	4.32E-06	Lipopolysaccharide biosynthesis protein
	*A6O24_14495*	2.52	2.28E-16	Carbohydrate kinase
	*A6O24_05830*	1.55	1.14E-06	Outer membrane lipoprotein carrier protein LolA
	*A6O24_07250*	3.11	2.53E-43	Channel protein TolC
	*A6O24_15615*	6.226	1.79E-54	ABC transporter protein
	*A6O24_00390*	2.23	6.60E-18	Tol Pal system beta propeller repeats protein TolB
	*A6O24_17005*	2.21	9.85E-08	Chemotaxis protein CheV
	*A6O24_16985*	2.99	2.05E-16	Chemotaxis protein CheA
	*A6O24_19210*	2.53	1.39E-23	Thioredoxin
	*A6O24_07465*	1.58	1.10E-09	Glutamine synthetase

The downregulated KEGG metabolic pathway was significantly enriched in the first seven pathways: enriched in the M pathway include sulfur metabolism (17), oxidative phosphorylation (23), pentose phosphate pathway (8), starch and sucrose metabolism (8); enriched in the CP pathway include flagellar assembly (16), bacterial chemotaxis (9); the GIP pathway is protein processing in the endoplasmic reticulum (4) (Figure 5D).

### 3.4 Analysis of genes related to biofilm formation

DEG analysis indicates that there are two key genes regulating QS, five genes related to biofilm formation, and six genes related to bacterial adhesion in *A. thiooxidans*. The QS communication system, mediated by N-acyl-homoserine lactone molecules, regulates biofilm formation (Díaz et al., 2021). SAM and acyl carrier proteins synthesize signaling molecules under the action of N-acyl-homoserine lactone synthase (*afeI*). At the same time, we detected that acyl carrier proteins and *afeI* were significantly upregulated 42 times and SAM protein increased by over 2 times. Similarly, detecting the *afeI* gene during the transcription process of *A. ferrooxidans* revealed that the expression levels of the *afeI* gene were higher in cells grown in sulfur and thiosulfate media compared to those grown in iron (Farah et al., 2005). In addition, the significantly upregulated genes detected include channel protein TolC, histidine kinase, and histidine phosphate transaminase (HolPase). TolC belongs to the outer membrane proteins of the type I secretion system (T1SS); common secretions include RTX toxins (Linhartová et al., 2010), HIyA, RtxA, proteases, lipases, and S layer proteins. HolPase is the second to last step catalyzing the biosynthesis of histidine (le Coq et al., 1999). The Tol system, also designated as Tol Pal, serves as a stabilizing factor within the outer membrane of Gram-negative bacteria (Bonsor et al., 2009). CheV is significantly upregulated in *A. thiooxidans*. It interacts with chemoreceptors and CheA and acts as a docking protein similar to CheW, potentially playing a role in signal transduction adaptation (Ortega and Zhulin, 2016). Previous studies have shown that chemotaxis is commonly present in mobile bacteria and also participates in various biological processes such as biofilm formation, auto–aggregation, and bacterial adhesion (Huang et al., 2019). Based on the QS regulators, Y3 has the potential to promote biofilm formation, adsorb sulfur sheets, and engage in bioleaching.

### 3.5 Analysis of molecular docking

The binding energies between AfeI and the ligand small molecules such as natural Y1 and artificial Y3 are −6.0 kcal/mol and − 6.5 kcal/mol, respectively (**Supplementary Table S5**). Normally, if the binding energy between the ligand and the target protein is less than −5, the ligand and the receptor protein can bind stably.

**Figure 6:**
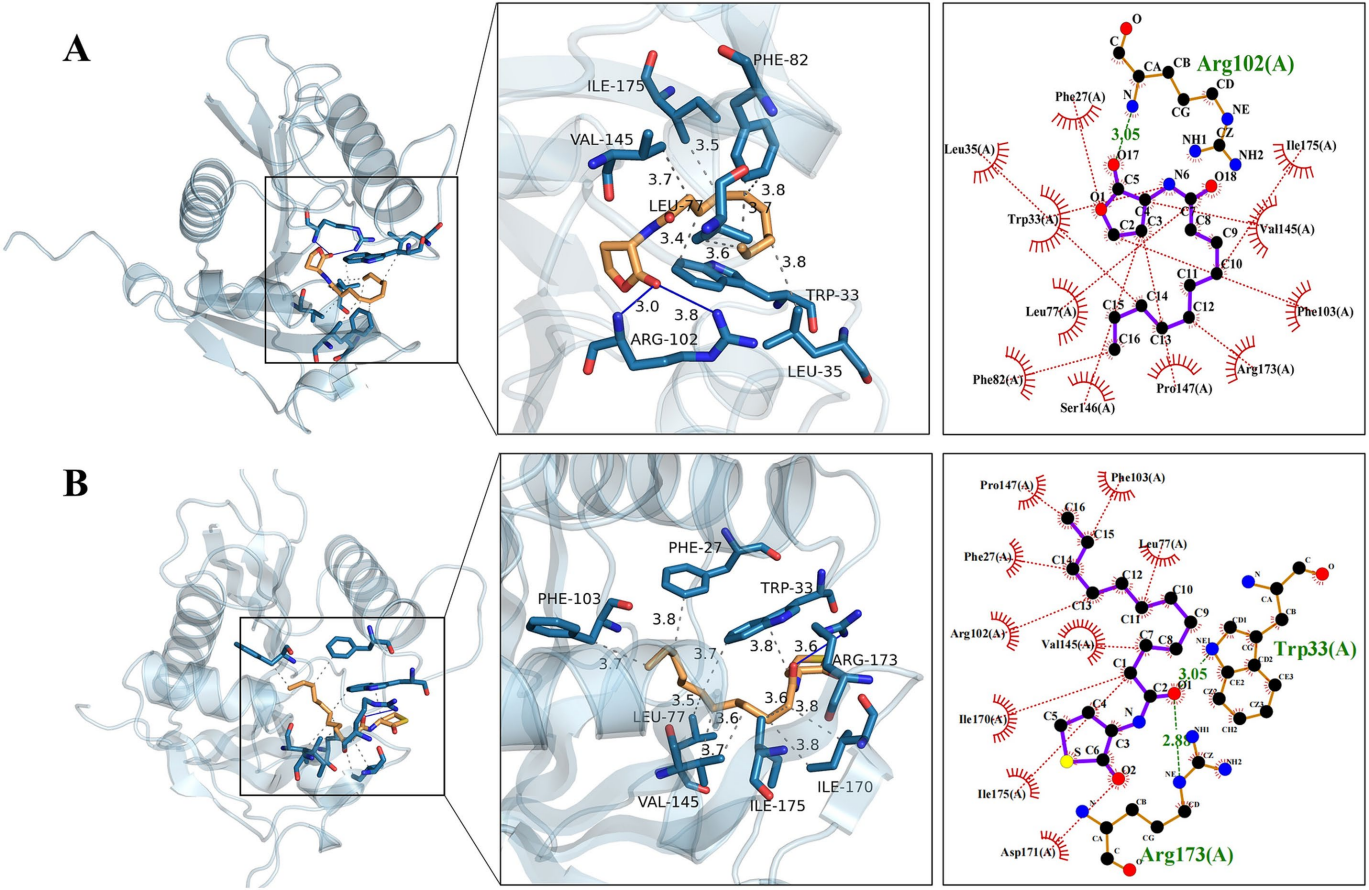
Natural Y1 and artificial Y3 are observed to dock with the AfeI protein. **(A)** The three-dimensional and two-dimensional details of the docking between Y1 and the AfeI protein are shown from left to right. **(B)** The three-dimensional and two-dimensional details of the docking between Y3 and the AfeI protein are shown from left to right. In the two-dimensional representation, green indicates hydrogen bonds, while red indicates hydrophobic interactions.

According to the three-dimensional diagram, Y1 and Y3 have similar spatial structures and bind to the AfeI protein through hydrogen bonding and hydrophobic interactions. However, Y1 has 11 residues that interact through hydrophobic forces, whereas Y3 has only 9 residues that interact through hydrophobic forces. In addition, Y3 can bind to the receptor molecule AfeI at the Arg173 and Trp33 sites through hydrogen bonding of two amino acid residues. However, Y1 has only one hydrogen-bonding residue at the Arg102 site. Y1 and Y3 share six common interacting residue sites: Arg-102, Val-145, Ile-175, Asp-171, Phe-103, and Leu-77. The residues of these conserved sites may play an important role in the binding between natural ligands and AfeI. In addition, similar results were obtained from the two-dimensional force analysis (Figure 6). It is precisely because of the differences in the number of interacting residues and forces mentioned above that the ligand small molecule Y3 can bind more stably to AfeI.

## 4 Discussion

The structure of AHLs allows for the postulation of several hypotheses, including modifications to the lactone ring and the AHL acyl side chain, or alterations to both simultaneously (Welsh and Blackwell, 2016; Chbib, 2020). Y1 retains the high serine lactone ring, preventing hydrolysis of AHL by transforming its lactone ring into a homocysteine lactone, resulting in Y2 (Welsh and Blackwell, 2016). Long chain AHL has been shown to be beneficial for biofilm formation (González et al., 2013). Therefore, Y3 is a better representative molecule for meeting the requirements of activity and preventing QQ enzyme degradation by simultaneously extending acyl side chains and changing the lactone ring structure.

**Figure 7:**
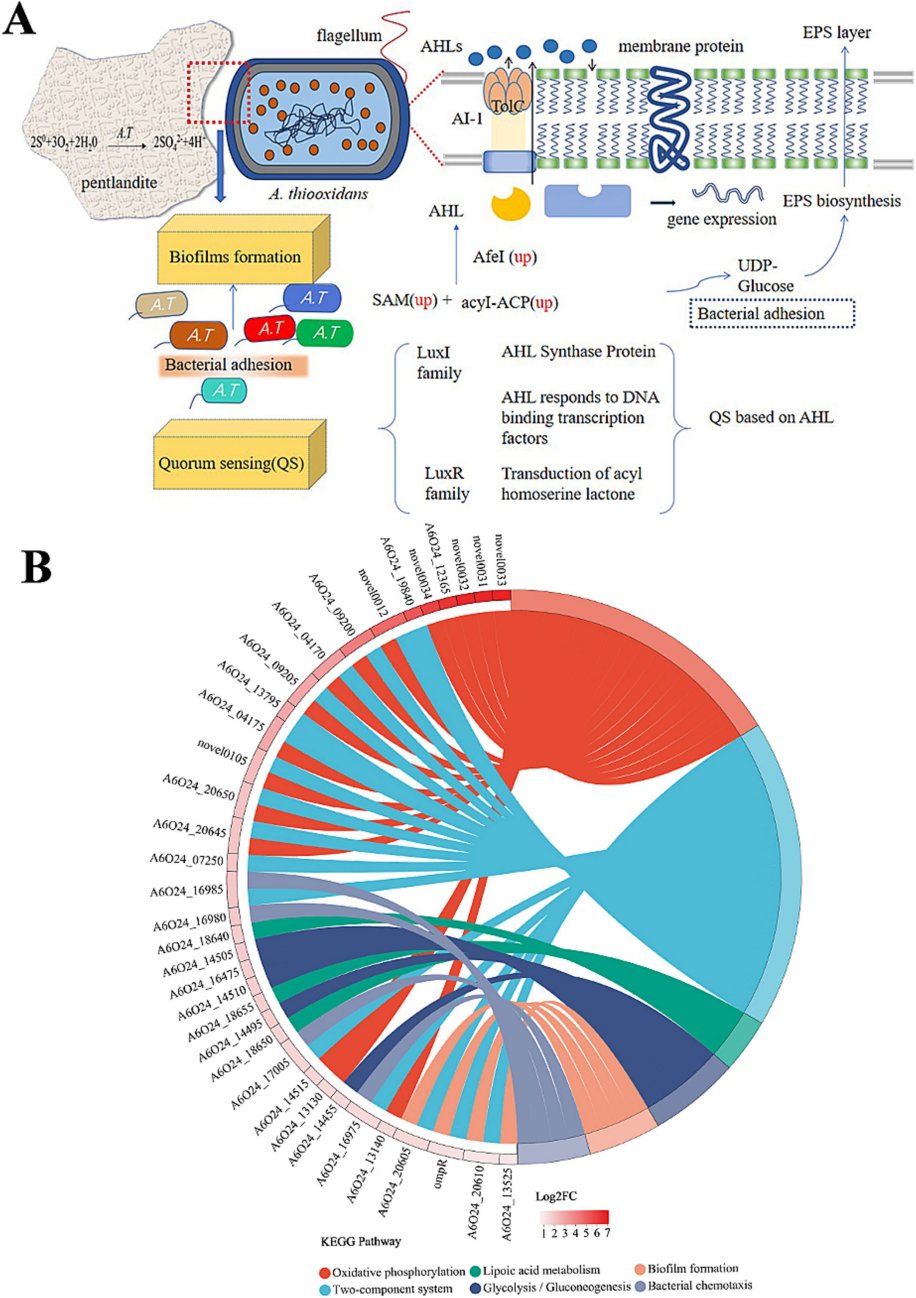
QS regulation mechanism and KEGG pathway information on the significantly enriched differentially expressed genes on the right side. **(A)** The red dashed line represents the enlarged image here, and genes marked as up represent significantly upregulated genes. **(B)** The chord diagram displays KEGG pathway enrichment, indicating pathways that are significantly enriched for differentially expressed genes. The genes on the left are arranged in descending order of log2FC.

In QS, bacteria synthesize a small molecule or short peptide signal that is either secreted or diffused out of the bacterial cell. This signal enables bacteria to communicate and coordinate their behavior with each other (Gerdt et al., 2015). TolC has multiple functions, one of which is being part of the type I secretion system (T1SS). Bacteria require the secretion of specific proteins and other molecules into the extracellular space to facilitate the acquisition of nutrients, the formation of biofilms (adhesins), or the invasion of hosts (Kanonenberg et al., 2018). The substrates for T1SS are proteins, and there is a recognition sequence in the amino acid sequence for transport. T1SS is comprised of TolC upregulated 3.1 times, an ABC transporter upregulated 6.2 times (Table 1), and a membrane fusion protein (MFP) that showed no significant difference expression. AHLs diffuse into and out of bacterial cells, and as the population of bacteria increases, so does the concentration of AHLs. Once the AHL concentration reaches a threshold level, it acts as a co-inducer, typically by activating LuxR-type transcriptional regulators to induce target gene expression (Yates et al., 2002) (Figure 7A).

Extracellular polymers (EPs) play an important role in strengthening cell attachment. The difference between polysaccharides and proteins in EPs causes the affinity difference between cells and the substrate (Xia et al., 2013; Li et al., 2016), which may be one of the possible reasons for the adsorption difference caused by ligands or simulated self-inducers. At the same time, EPs also affect bacterial attachment and biofilm formation (González et al., 2013). The *pel* genes are positively regulated by AHLs, which participate in the molecular network for PEL exopolysaccharide biosynthesis by *A. thiooxidans* (Díaz et al., 2018). Surprisingly, *pel*-related genes were not changed in this experiment. The synthesis of PEL exopolysaccharides may be subject to regulation by several yet unidentified signaling molecules. It is widely understood that the attachment of *A. thiooxidans* is linked to the creation of an active biofilm. Our experimental results found that after the action of analog Y3 on *A. thiooxidans*, carbohydrate kinase and lipopolysaccharide biosynthesis protein were upregulated separately by 2.5 and 1.9 times which also confirms that Y3 can improve adhesion ability to the bacteria, yet there was no obvious change in protein content. The study investigates the function of OmpR, a two-component system (TCS) response regulator. A mutation in OmpR led to a decrease in biofilm activity and an increase in bacterial motility (Figure 7B). In addition, the recombinant OmpW protein was found to restore biofilm activity and increase the content of extracellular polysaccharides (Ma et al., 2023). These results suggest a direct relationship between OmpR and biofilm formation and motility. TCS is the cellular signaling circuit, which first emerged in the early 1980s, particularly through its discovery in the model bacterium *Escherichia coli* (Papon and Stock, 2019). Detection results found that *A6O24_16560* was closely related in the process of EIP, upregulated 1.75 times (Table 1). It is one of the most important participants in bacterial and archaeal signal transduction (Zschiedrich et al., 2016). Multiple regulatory mechanisms are speculated to exist in *A. thiooxidans*, specifically TCS. It should be noted that the chemically synthesized analog Y3 may have a synergistic effect on both the QS system and TCS simultaneously (Figure 7).

Ten extreme microorganisms belonging to *Thiobacillus* were compared, and *A. thiooxidans* had two thiooxide proteins (Zhang et al., 2016a). The comparative analysis indicates that strain-specific genes may play a role in adapting to the leaching environment within the permitted range. In addition, the *sor* gene exists in *A. thiooxidans,* and the protein SOR plays a key role in the catalytic oxidation of elemental sulfur and RISC (Zhang et al., 2015). One thioredoxin (*A6O24_19210*) was upregulated 2.53 times in this experiment. Genetic movement genes were acquired through horizontal gene transfer during mining (Travisany et al., 2014; Zhang et al., 2016b). The cascade signaling of TCS provides important clues for the evolutionary transition from prokaryotes to eukaryotes (Chauhan, 2015). Therefore, it is worth exploring whether a similar phenomenon occurs in *A. thiooxidans*.

## 5 Conclusion

The design and synthesis of Y3 have been demonstrated to promote biofilm formation and adhesion ability and improve bioleaching efficiency better than natural signaling molecules. The addition of Y3 upregulated the *afeI* gene for QS and catalytic signal molecule synthesis in *A. thiooxidans*. The chemical synthesis of the QS agonist offers a useful approach to address the low efficiency of biological leaching. It also enhances our knowledge of quorum communication and multi-system regulation among leaching microorganisms.

## Data Availability

The datasets presented in this study can be found in online repositories. The names of the repository/repositories and accession number(s) can be found at the following website: https://www.ncbi.nlm.nih.gov/BioProject: PRJNA1082031.
