# Supplementary material for: Design and synthesis of quorum-sensing agonist for improving biofilm formation and the application of Acidithiobacillus thiooxidans in bioleaching
Source: Front Microbiol. 2024 Oct 15;15:1465633. doi: 10.3389/fmicb.2024.1465633 (PMC11519741; doi:10.3389/fmicb.2024.1465633)
Supplement: Supplementary file 1 [file Data_Sheet_1.pdf]

Supplementary Material

1 Supplementary Figures

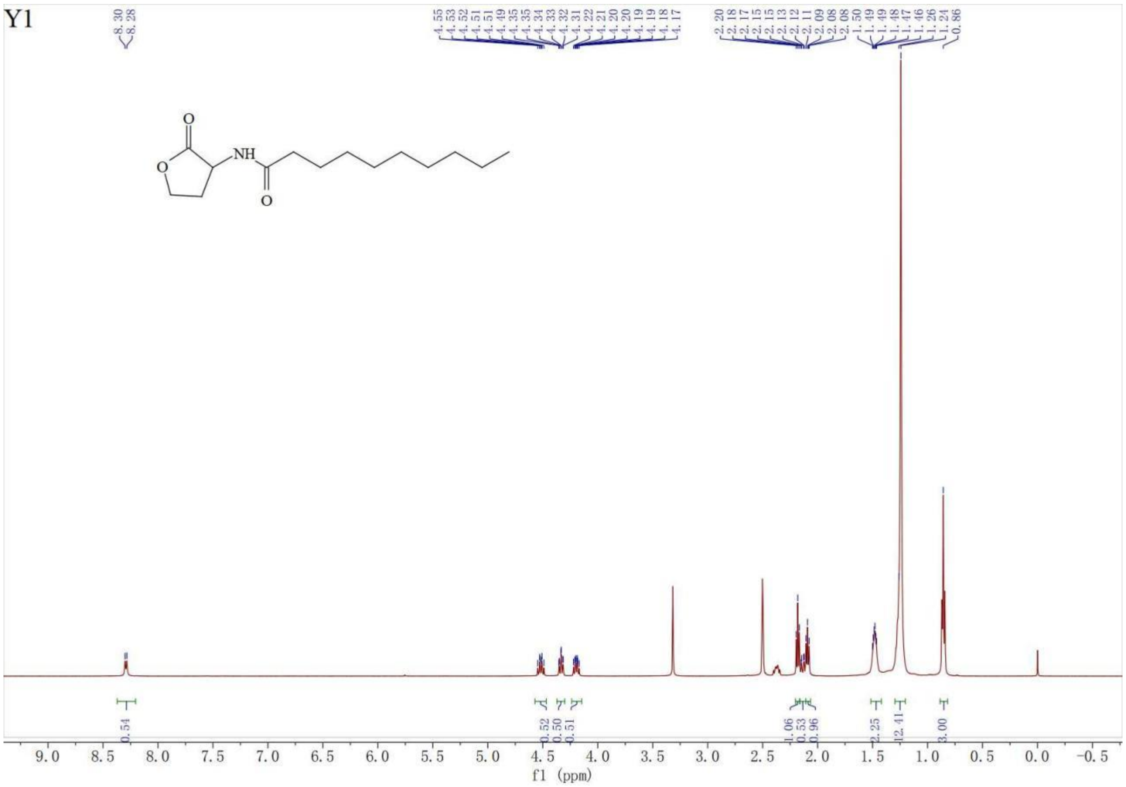

Supplementary Figure S1. The ^{1}H-NMR (500MHz, DMSO-d6) of Y1.

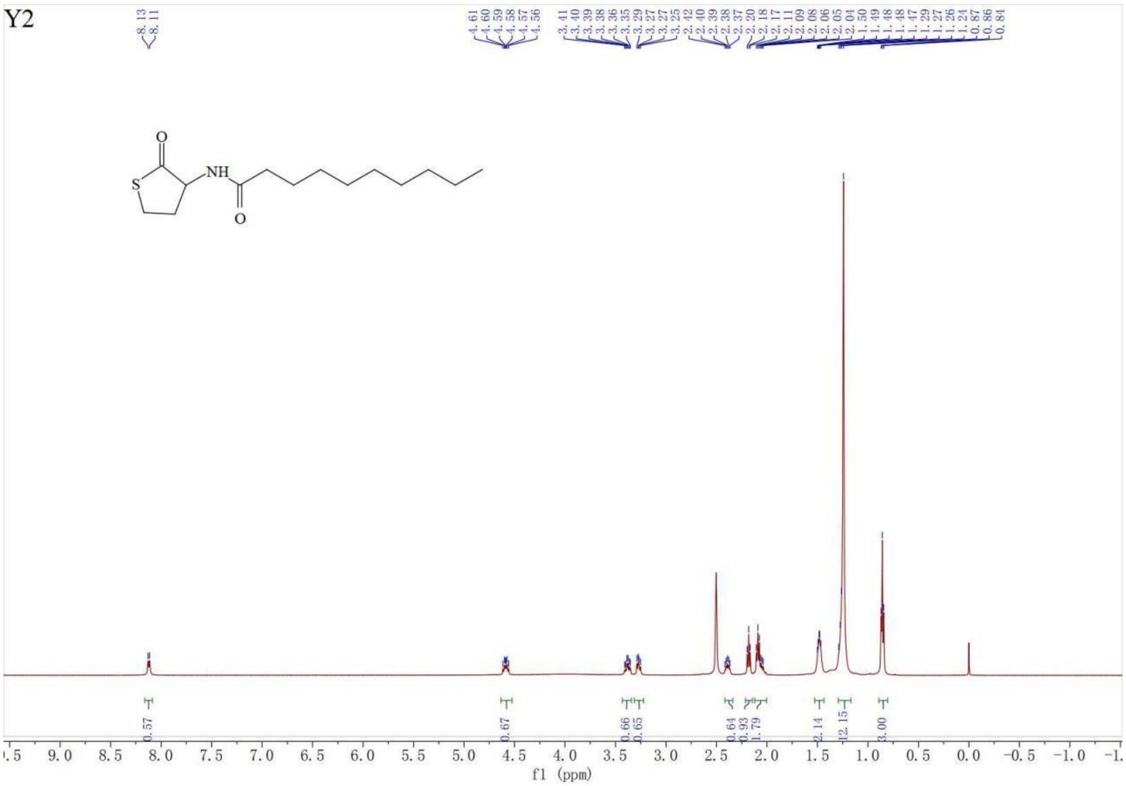

Supplementary Figure S2.1H-NMR (500MHz, DMSO-d6) of Y2.

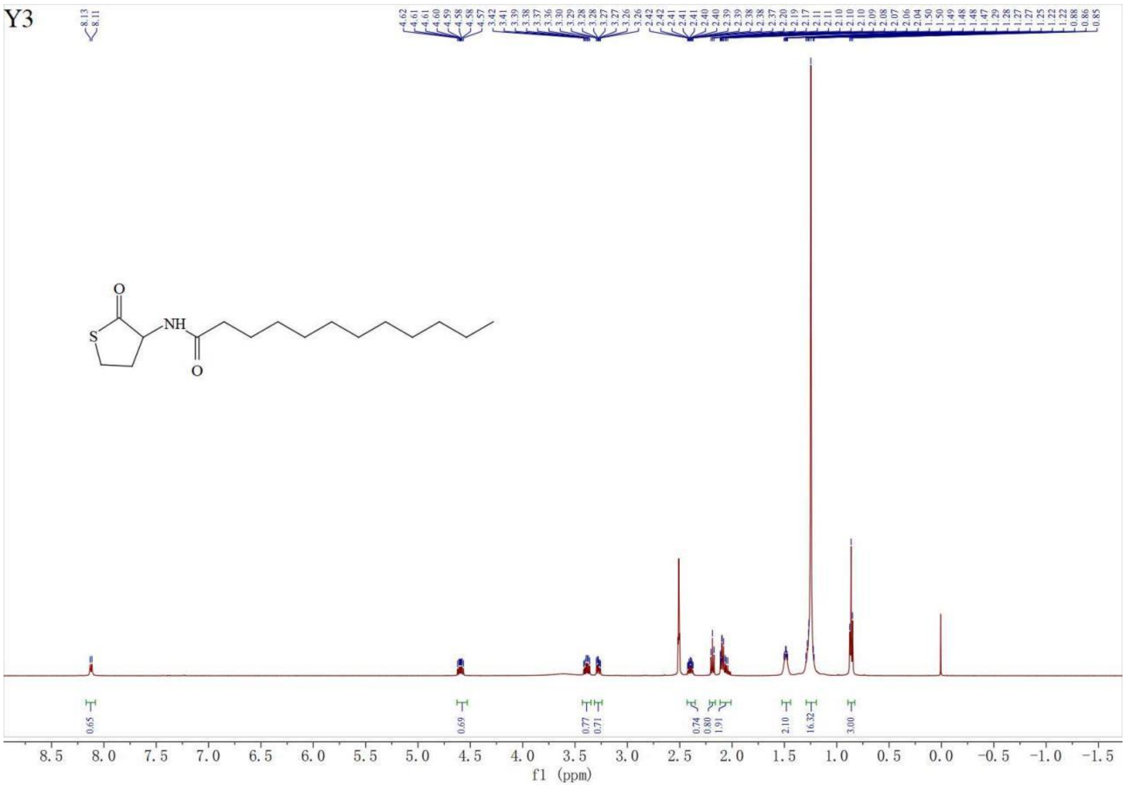

Supplementary Figure S3. The 1H-NMR (500MHz, DMSO-d6) of Y3.

2 Supplementary Tables

Supplementary Table S1 Langmuir and Freundlich fitting adsorption parameters under different treatments

Strains	Adsorbed substrate	Y3	Langmuir fitting parameters			Freundlich fitting parameters		
			$K_A \times 10^{-10}$	$X_M \times 10^{8}$	R^2	K_F	n	R^2
A.thiooxidans	Sulfur pieces	CK	5.3	4.5	0.9842	1.00E-42	0.17	0.9706
		50μM	5.7	3.0	0.9748	1.00E-54	0.14	0.9849
		150μM	5.8	3.5	0.9867	1.00E-42	0.17	0.9895
		300μM	6.1	3.2	0.9862	1.00E-35	0.21	0.9617

Supplementary Table S2 Total RNA detection results

Number	Sample	Concentration (ng/μL)	Total (μg)	OD260/280	OD260/230	RQN
1	CK_1	609	21.32	2.05	2.27	9.3
2	CK_2	630.2	22.06	2.04	2.28	9.4
3	CK_3	653.8	22.88	2.04	2.3	9.7
4	Y3_1	524.9	18.37	2.06	2.19	8.6
5	Y3_2	484.6	16.96	2.07	2.25	8.6
6	Y3_3	511.1	17.89	2.06	2.23	8.4

Supplementary Table S3 Transcriptome Sequencing Data

Reads		Mean read No.	Mean base No.	Error Rate (%)	Mean Q20 (%)	Mean Q30 (%)
Raw	CK-1	33619170	5076494670	0.0143	96.97	92.62
	CK-2	35507622	5361650922	0.0139	97.24	93.08
	CK-3	37562990	5672011490	0.0142	96.95	92.84
	Y3-1	36956542	5580437842	0.0166	95.34	89.7
	Y3-2	35639276	5381530676	0.0178	94.92	88.2
	Y3-3	39993546	6039025446	0.0152	96.33	91.43
Clean	CK-1	33221444	4683683791	0.0127	98.22	94.78
	CK-2	35105688	4968754239	0.0126	98.36	95.06
	CK-3	37150400	5243727490	0.0125	98.41	95.22
	Y3-1	36428176	4982822675	0.0138	97.42	93.22
	Y3-2	35065374	4773163744	0.0147	96.98	91.75
	Y3-3	39485026	5484838839	0.0131	97.91	94.18

Supplementary Table S4 Statistics of Sequencing Data Comparison Results

Sample Name	Total Reads	Genome Mapped		Unmapped		Uniq Mapped	
		Reads	Reads Ratio(%)	Reads	Reads Ratio(%)	Reads	Reads Ratio(%)
CK-1	33221444	20936606	63.02	12284838	36.98	20365714	61.3
CK-2	35105688	22250433	63.38	12855255	36.62	21613203	61.57
CK-3	37150400	24287247	65.38	12863153	34.62	23587494	63.49
Y3-1	36428176	23751919	65.2	12676257	34.8	23086386	63.38
Y3-2	35065374	23849836	68.02	11215538	31.98	23177130	66.1
Y3-3	39485026	29407892	74.48	10077134	25.52	28638775	72.53

Supplementary Table S5 Binding sites and docking fractions of ligand Y1 andY3 with AfeI

Ligand	Y1	Y3
Docking score (Kcal/mol)	−6.0	−6.5
Hydrogen bond-interacting residues	Arg-102	Arg-173，Trp-33
Of the hydrophobic-acting force residues	Phe-82, Phe-103, Leu-77, Leu-35, Arg-102, Arg-173, Val-145, Trp-33, Ser-146, Asp-171, Ile-175	Pro-147, Phe-103, Phe-27, Leu-77, Arg-102, Val-145, Ile-170, Ile-175, Asp-171
